# Supplementary material for: Complexome profiling of the Chlamydomonas psb28 mutant reveals TEF5 as an early PSII assembly factor
Source: Plant Cell. 2025 Mar 17;37(6):koaf055. doi: 10.1093/plcell/koaf055 (PMC12164590; doi:10.1093/plcell/koaf055)
Supplement: koaf055_Supplementary_Data [file koaf055_supplementary_data.zip › psb28_interactive.zip!/plots/intensity/qualityPlots/peptides/COX2A_Cre03.g154350.t1.2.html]

### Cre03.g154350.t1.2

Trivial name: COX2A  
SeqCoverage [median]: 6.70  
Mass: 30.68 kDa  
  
 

| Sequence | MissedCleavage | Mass [Da] | wtIntensity | muIntensity | Score |
| --- | --- | --- | --- | --- | --- |
| LLDPDR | 0 | 727.39 | 3197.78 | 3019.11 | 97.60 |
| LLDPDRLVGIAEK | 1 | 1437.82 | 129011.31 | 357224.10 | 89.96 |
| LVGIAEK | 0 | 728.44 | 9459.84 | 4948.09 | 100.74 |
| VLKPEK | 1 | 712.45 | 0.00 | 5695.24 | 138.48 |
|  |  |  | 141668.9 | 370886.5 |  |
